# Evaluation of different fiducial markers for image-guided radiotherapy and particle therapy

**DOI:** 10.1093/jrr/rrt071

**Published:** 2013-07

**Authors:** Daniel Habermehl, Katrin Henkner, Swantje Ecker, Oliver Jäkel, Jürgen Debus, Stephanie E. Combs

**Affiliations:** 1 Department of Radiation Oncology, University Hospital of Heidelberg, Im Neuenheimer Feld 400, 69120 Heidelberg, Germany; 2 Heidelberg Ion Beam Therapy Center (HIT), Im Neuenheimer Feld 450, 69120 Heidelberg, Germany

**Keywords:** carbon ion therapy, liver tumors, fiducial marker, raster-scanning technique, moving organs

## Abstract

Modern radiotherapy (RT) techniques are widely used in the irradiation of moving organs. A crucial step in ensuring the correct position of a target structure directly before or during treatment is daily image guidance by computed tomography (CT) or X-ray radiography (image-guided radiotherapy, IGRT). Therefore, combinations of modern irradiation devices and imaging, such as on-board imaging (OBI) with X-rays, or in-room CT such as the tomotherapy system, have been developed. Moreover, combinations of linear accelerators and in-room CT-scanners have been designed. IGRT is of special interest in hypofractionated and radiosurgical treatments where high single doses are applied in the proximity of critical organs at risk. Radiographically visible markers in or in close proximity to the target structure may help to reproduce the position during RT and could therefore be used as external surrogates for motion monitoring. Criteria sought for fiducial markers are (i) visibility in the radiologic modalities involved in radiotherapeutic treatment planning and image guidance, such as CT and kilovoltage (kV) OBI), (ii) low production of imaging artifacts, and (iii) low perturbation of the therapeutic dose to the target volume. Photon interaction with interstitial markers has been shown to be not as important as in particle therapy, where interaction of the particle beam, especially with metal markers, can have a significant impact on treatment. This applies especially with a scanned ion beam. Recently we commenced patient recruitment at our institution within the PROMETHEUS trial, which evaluates a hypofractionation regime, starting with 4 x 10 Gy (RBE), for patients with hepatocellular carcinoma. The aim of this work is, therefore, to evaluate potential implantable fiducial markers for enabling precise patient and thus organ positioning in scanned ion beams. To transfer existing knowledge of marker application from photon to particle therapy, we used a range of commercially available markers of different forms and sizes, consisting of carbon and gold materials, and evaluated them for their potential use in the clinical setup with scanned ion beams at our institution. All markers were implanted in a standardized Alderson phantom and were examined using CT scans and orthogonal kV OBI in our clinical routine protocol. Impact on beam perturbation downstream of the markers in the plateau region of a spread-out Bragg peak (SOBP) was estimated by using radiographic films for clinical proton and carbon ion beams of high and low energies. All tested markers achieved good visibility in CT and kV OBI. Disturbances due to artifacts and dose perturbation were highest in the arbitrarily folded gold and the thickest gold marker, but especially low in the carbon marker. Dose perturbation was highest in the arbitrarily folded gold marker. In summary, the analyzed markers offer promising potential for identifying target structures in our treatment setup at HIT and will soon be used in clinical routine. However, a careful choice of marker, depending on the tumor localization and irradiation strategy, will need to be made.

## INTRODUCTION

Modern radiotherapy has undergone enormous improvements over recent years, resulting in higher dose conformity by using intensity-modulated radiation techniques, particle therapy (PT) with charged ions, and image guidance [1]. Therefore, combinations of modern irradiation devices and imaging, such as on-board imaging (OBI) with X-rays, or combinations with in-machine computed tomography (CT) such as the tomotherapy system have been developed. Moreover, combinations of linear accelerators and in-room CT-scanners have been designed. The clinical implementation of raster-scanned ion beam therapy for a broad range of oncologic diseases has been successfully carried out at the Heidelberg Ion-beam Therapy Center (HIT) since 2009 [2, 3]. Most patients in that institution receive particle treatment in cases of brain, skull base and sacral tumors, while treatment for prostate, liver and pancreatic cancer has recently started. The current clinical trial PROMETHEUS at our institution examines a hypofractionation protocol with a dose-escalation strategy for patients with hepatocellular carcinoma [4, 5]. Treatment planning and irradiation of hepatic tumours warrants the consideration of organ and tumor motion in all six spatial directions, and elaborate treatment-planning procedures [6]. The internal target volume (ITV), and thus the planning target volume (PTV), include macroscopic and microscopic tumor spread, as well as setup uncertainties and organ motion throughout the breathing cycle. The visualization and morphometric localization of hepatic tumors in treatment setup without contrast-enhanced imaging is a considerable challenge. While orthogonal kV OBI allows comparison and estimation of bony tissue anatomy of the patient and underlying CT data, it is of great importance to include information about (static) soft tissue, and especially about the range of organ motion in a defined immobilization setup. One solution to this problem is the insertion of radio-opaque fiducial marker implants in the observed organ or in the tumor tissue itself. Most commercially available fiducial markers are made of high-Z materials, which cause artifacts in conventional CT scans, thus perturbing the calculated dose to the surrounding tissue due to an incorrect representation of the electron density near the marker [7–10]. Most notably in the case of PT, precise location of the target volume is crucial for correct dose distribution because of the steep dose gradient resulting from the spread-out Bragg peak (SOBP). Fiducial markers should therefore exhibit the following features: visibility in the required imaging modality (e.g. CT scans, magnetic resonance imaging, kV OBI), absence of (or at least few) artifacts, easy application, and sufficient immovability after insertion.

This work describes the evaluation of different commercially available markers for patients with tumors in movable organs.

## MATERIAL AND METHODS

We evaluated the following commercially available markers: Visicoil (IBA Dosimetry GmbH, Schwarzenbruck, Germany), Beammarks (Beampoint AB, Kista, Sweden), Gold Anchor (Naslund Medical AB, Huddinge, Sweden), Carbon Marker (CIVCO Medical Solutions, Kalona, IA, USA) and BiomarC (Carbon Medical Technologies, MN, United States). The markers were provided by the manufacturers. Descriptions and physical characteristics are summarized in Table 1.

**Table 1.** Description of the different fiducial markers evaluated

Name	Manufacturer	Material	Shape	Length in mm (vendor information)	Diameter in mm (vendor information)
Visicoil	IBA	gold	coil-shaped	20	1.1; 0.75; 0.5 and 0.35
Beammarks	Beampoint AB	nitinol	star-shaped	5	1.2
Gold Anchor	Naslund Medical AB	gold	straight or folded	200 and 100	0.27 (measured at HIT)
Acculoc Carbon Marker	Carbon Medical Technologies (vendor: CIVCO)	zirconium oxide covered by pyrolytic carbon	bone-shaped	3	1
BiomarC	Carbon Medical Technologies (vendor: Vigeo)	zirconium oxide covered by pyrolytic carbon	bone-shaped	3	1

In terms of CT imaging and artifacts, visibility in orthogonal kV OBI, proton and carbon ion beam field perturbation and water-equivalent path length (WEPL) were investigated. Furthermore, we evaluated the markers according to their usability for our standard workflow of patient treatment at HIT, which includes use of digitally reconstructed radiographs (DRRs) of CT images for matching with kV OBI.

Not all markers were analyzed to the same degree due to availability and expected relevance to the results.

### CT imaging

**Fig. 1.**
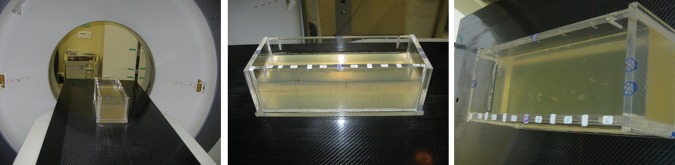
Rectangular box filled with (manufactured) gelatin solution, in which different markers are inserted. Setup for CT scan under standardized conditions (left); box from different views (middle and right).

First, markers were implanted within a rectangular box filled with a homogenous gelatin solution. Markers were implanted according to the manufacturer's instructions with prepared application needles (Fig. 1). Second, three of the above-mentioned markers were implanted into a standardized Alderson phantom, roughly in the liver position. To simulate the designated protocol, kV CT scans were carried out using a Somatom Sensation 4 (Siemens) according to our in-house standardized treatment-planning body protocol with 120 kV, 300 mAs, 500-mm field of view and 3-mm slice thickness. Transversal pixel resolution was 0.977 mm.

### kV OBI

At HIT, an OBI system has been installed for patient position verification prior to treatment. Markers implanted in tumors will be used for evaluating position and movement of a tumor. Together with information on the position of bony structures, the markers might be used to match orthogonal X-ray images and DRRs, since the position of both influences the range and dose distribution of ion beams. Using this same setup, orthogonal X-ray imaging and OBI are both performed at HIT. The OBI system is a product of Siemens Medical Healthcare and is installed in all three treatment rooms at HIT.

### DRRs

In the standard procedure for patient position verification at HIT, orthogonal X-ray images are compared with the DRRs derived from the planning CT. Hence, markers need to be able to be well defined in DRRs and thus used as a target for position verification. DRRs obtained purely from CT images of markers in the Alderson phantom simulating the situation in a patient, were generated within the workflow of patient treatment in the position verification step of the treatment-application system. Hence, a treatment plan for the Alderson phantom was generated within the treatment-planning system currently used at HIT (Syngo PT Planning, Siemens, Germany).

### Evaluation of downstream perturbation of carbon and proton ion beams

Megavoltage CT markers were placed downstream of 20 mm PMMA on a 1-mm PMMA plate, which was followed by a KODAK X-OmatV film and further PMMA plates. Four films were irradiated, each with an homogeneous field of 1 Gy of carbon ion beam energies of 150.42MeV/u and 309.75MeV/u, and proton beam energies of 80.9MeV and 202.14MeV. Thus, the markers were placed in the proximal region. Otherwise, by placing the markers in the Bragg peak region the beam would have stopped within the markers. For carbon ions a ripple filter was used.

### Estimation of WEPL

To estimate the impact on ion beam range calculation, the WEPLs of the different markers were compared. For gold the WEPL was measured at HIT, where it is routinely used. For both the nitinol and the carbon markers, the WEPLs were estimated purely by comparing their chemical compositions. The Beammarks was assumed to consist of Ni and Ti only.

## RESULTS

**Fig. 2.**
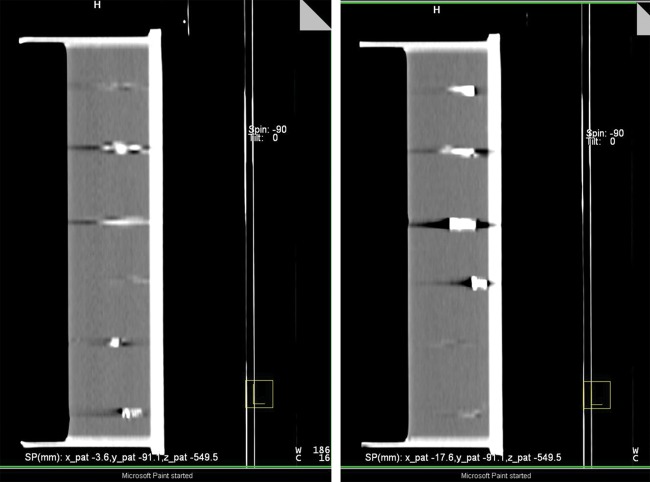
Sagittal plane of markers in gelatine solution, from top to bottom: Visicoil: 0.35 x 20 mm; Visicoil: 0.75 x 20 mm; Visicoil: 1.1 x 20 mm; Gold Anchor: 0.27 x 20 mm, Beammarks: 1.2 x 5 mm; Gold Anchor: 0.27 x 20 mm.

Visibility in gelatin solution was tested as prescribed in the *Material and Methods* section. A representative sagittal screenshot through the applied markers in the experimental setting is presented in Fig. 2. All markers (Visicoil: 0.35 x 20 mm; 0.7 5 x 20 mm; 1.1 x 20 mm; Gold Anchor: 0.27 x 20 mm; Beammarks: 1.2 x 5 mm) were visible, and produced different intensities of artifacts. The Gold Anchor marker was included twice because different arbitrary folding behaviors were used when it was getting inserted into the gelatin solution.

**Fig. 3.**
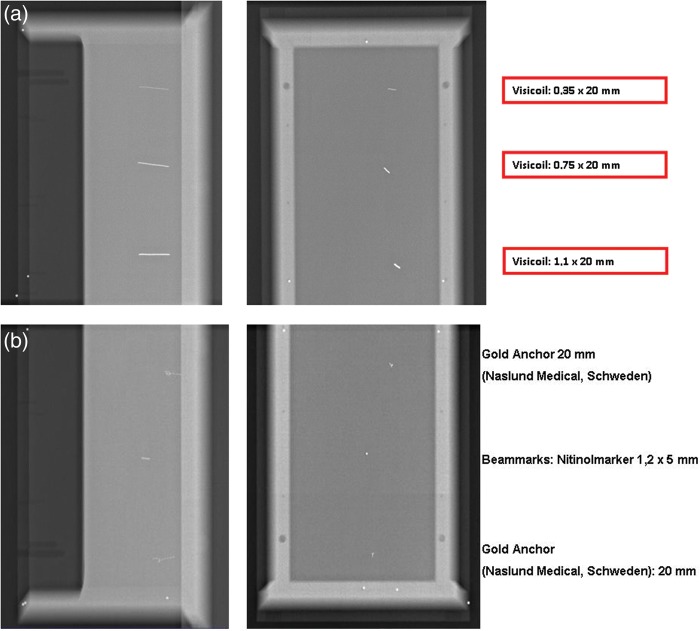
CT scan of different markers in gelatin solution. Front section (**a**) containing three different sized Visicoil markers (sizes from top to bottom: 0.35 x 20 mm, 0.75 x 20 mm, 1.1 x 20 mm). Back section (**b**) containing Gold Anchor marker (0.27 x 20 mm) in first and last position and Beammarks (1.2 x 5 mm).

Visibility of the inserted markers in this setup was also examined using the OBI kV imager in a HIT treatment room (Fig. 3). For this purpose, orthogonal (posterior-anterior and lateral left-right) radiographs of the box were generated. Again, all markers achieved good visibility, with the best results for the Visicoil markers, increasing with thickness (0.35 mm, 0.75 mm, 1.1 mm).

**Fig. 4.**
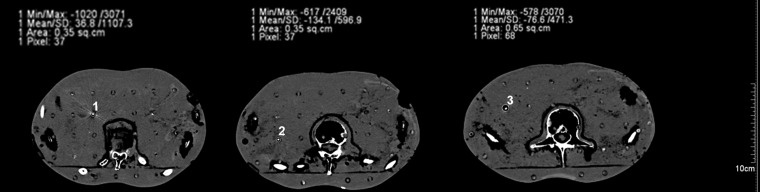
Three markers were placed in the Alderson phantom, Visicoil (0.35 x 20 mm), Beammarks (1.2 x 5 mm) and the Carbon marker (1 x 3 mm). On the left upper parts maximal and minimal HU (Hounsfield Units) values of the marker surrounding regions are presented.

Further testing was performed using a standardized Alderson phantom to analyze visibility in the more realistic setup of surrounding anatomic structures. Three markers (Visicoil: 0.35 x 20 mm, Beammarks: 1.2 x 5 mm, and a CIVCO Carbon marker: 1 x 3 mm) were placed in the liver localization in the phantom, and subsequently a CT scan was performed according to our in-house standard protocol for HIT patients (Fig. 4). On the upper part of the images the measured minimum and maximum Hounsfield Unit (HU) values of the marker regions are demonstrated. The broadest HU-range is seen for the Visicoil marker, whereas the other two markers had smaller ranges. In particular, the Beammarks-marker had a relatively low maximal HU value of 2409 (compared with 3070 HU for the other markers).

**Fig. 5.**
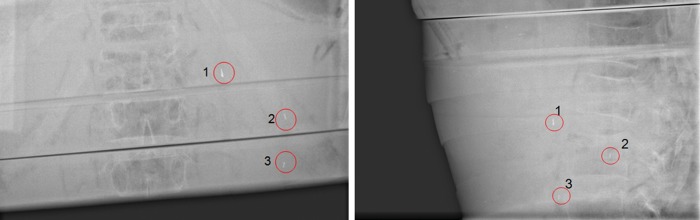
Orthogonal on-board kilovoltage imaging radiographs (OBI). Posterior-anterior (left) and lateral (right) view.

By using the prepared Alderson phantom with inserted markers in the above-mentioned positions, as described in Fig. 4, kV OBI was done (Fig. 5). All markers achieved good visibility again, even in a standardized phantom in a realistic treatment scenario.

**Fig. 6.**
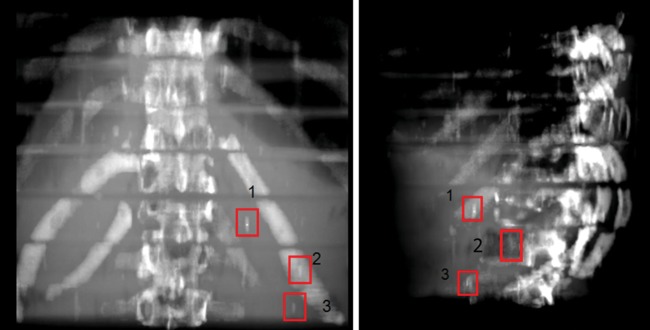
Digitally reconstructed radiographs (DRRs) of the previous CT scan (see Figs 4 and 5). Marker positions: 1 = Visicoil: 0.35 x 20 mm, 2 = Beammarks: 1.2 x 5 mm, 3 = Carbon marker, 1 x 3 mm.

Subsequently a DRR was generated from this dataset using the Siemens Syngo PT planning software (Siemens, Erlangen, Germany) (Fig. 6). Whereas all markers were visible in the posterior-anterior radiograph, the marker in position 2 (Beammarks) could not be discriminated in the lateral radiograph.

**Fig. 7.**
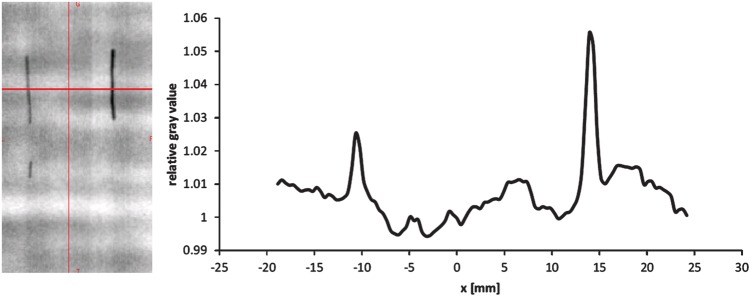
Gray value of film irradiation with 150.42 MeV/u carbon ions of Visicoil 0.75mm (upper left), Visicoil 1.1mm (upper right) and Beammarks (lower left) marker and the relative gray value of the two Visicoil markers.

The impact on ion beam field perturbation ∼1.65 mm behind the markers (site chosen because markers were placed on 1 mm of PMMA in front of the film) was estimated by evaluating the gray level of the films with Mephysto MC (PTW, Freiburg, Germany) [2]. In the proximal region of the Bragg peak at the highest proton and carbon ion beam energies, no remarkable influence on the gray level downstream of the markers was observed. For the small energies, we observed a gray level increase between up to 6% for the 1.1-mm Visicoil (Fig. 6) and 1.5% for the carbon-coated marker. For gold, the WEPL scaling factor used at HIT is 10.2. For nitinol the estimated WEPL scaling factor is between 3 and 5, and for carbon coated zirconium oxide it is around 3.

## DISCUSSION

Precise patient positioning is crucial for the application of scanned ion beams to ensure the calculated dose deposition. Image-guided radiotherapy (IGRT) usually involves cone-beam CT or orthogonal kilo- or megavoltage CT images, and allows an immediate pre-therapeutic comparison of the current patient anatomy with the treatment-planning CT scan or the DRRs [11]. At HIT, orthogonal kV OBI is the standard procedure before beam application, but this can be extended by an offline CT scanner with semi-automatic shuttle transportation to the treatment room [12].

This workflow was recently established for several indications and tumor sites, such as sacral chordoma and hepatocellular carcinoma, at our institution. In case of the latter, respiratory-dependent organ motion has to be taken into account in the treatment-planning procedure [13–15]. Patient positioning is evaluated by direct comparison of the treatment-planning CT scan with the CT scan made just before beam application. The exact tumor position may not be identified from the regular native scan. Therefore, application of fiducial markers in the tumor or the surrounding tissue may be beneficial for localization, but may also lead to higher dose perturbation because of the higher material density, especially in cases where the required beam direction(s) cannot be anticipated at an early time-point in the planning procedure. In the case of moving organs such as liver and the lungs, and to a lesser extent the prostate and pancreas, several elaborate radiation techniques may be chosen, such as gating, re-scanning and tracking [16].

In our analysis of a range of fiducial markers, we focused on eligibility for inclusion in the clinical workflow at HIT. First, visibility in the OBI and discrimination from the surrounding tissues was considered crucial for any clinical implementation of an implantable marker. In our simulated treatment setting, all analyzed markers were visible in the homogenous gelatin solution, as well as when implanted in an Alderson phantom under ‘real-life’ conditions, and were therefore considered eligible for a clinical routine setting—in principle. Second, artifact production is a known side-effect of metallic implants and depends on the materials' Z-value. While on the one hand pure gold markers have an excellent visibility in different imaging modalities, even in deep intra-corporal positions and MV-CT imaging, on the other hand they produce disadvantageous artifacts in CT imaging. This has a direct impact on dose calculation and could potentially result in dose perturbation because of (i) differences in target-volume delineating in the proximity of the artifacts, and (ii) incorrect representation of the electron density and thus the HU surrounding the marker [10].

High-Z material contained in markers such as the Gold Anchor markers produce artifacts, and thus the above-mentioned problems are likely to occur. The evaluation of alternative materials, e.g. zirconium oxide (ZrO_2_)-core carbon-coated markers, revealed a relatively low incidence of artifacts, as seen by other research groups in comparable settings [17]. Furthermore, the carbon marker was clearly visible in kV OBI as well as in the DRRs, making this product a potential candidate for further clinical testing. Nitinol markers were designed to minimize artifacts while achieving high visibility,. In our analysis we had problems identifying the nitinol marker in the sagittal DRRs, but the main limitation was the localization of the marker at the coronal level of the vertebral column, which created a disadvantageous overlap of structures. Furthermore, the quality of a DRR depends on resolution in the *x*, *y* and *z* directions. Because of the (rather large) slice thickness of 3 mm in the CT scans, the volume effect has a considerable impact and thus smaller markers appear larger. A strategy to overcome this effect would be to choose a smaller slice thickness and thus achieve a higher resolution in the initial CT scan.

It is worth noting that the experimental setup does not take into account fiducial migration and consecutive dislocation when implanted in organs such as the liver, pancreas and prostate, as recently reported by Kim *et al*. [18, 19]. However, significant migration is a rare event and markers should be implanted in close proximity to the target volume for precise localization during treatment [19, 20]. These findings, though relevant, are beyond the scope of this work.

Markers had an influence on the fluence, dose distribution and range of ions in the studied cases. However, the marker material, its thickness and its position in the treatment field also affected the degree of influence. Thus, the 1.1- and 0.75-mm Visicoil markers had the biggest impact on dose due to the high Z and the large diameter compared to 0.5 and 0.35 mm. In terms of dose perturbation, the Beammarks was comparable to the 0.5-mm Visicoil marker. The BiomarC had the second smallest influence, and the 0.35-mm Visicoil marker had no influence on the dose (Fig. 7). Effects on absolute dose may be studied with Monte Carlo calculations.

Markers with low WEPL should have the smallest impact on range uncertainties. However, for gold, nitinol and carbon-coated zirconium oxide, the WEPL scaling factor cannot be expressed using the clinically used HU-WEPL calibration curve. This is because, the HU value for e.g. gold markers is already saturated and the curve is only valid for tissue-like materials [21]. Thus, to implement the Beammarks or the BiomarC marker in the treatment-planning system at HIT the WEPLs will need to be measured to assure a correct range calculation. Therefore, material samples will need to be provided by the vendors.

## CONCLUSION

In summary, the evaluated markers were thoroughly tested in our current clinical workflow. In all the important imaging techniques, including CT scans, kV-OBI and DRRs, visibility could be documented. However, none of the evaluated markers is ready to be used within the treatment field of ion beams. For use in photon beams the main criteria may be the artifacts produced in the planning CT, whereas for ion beams knowledge of the real WEPL is important too. Thus, at HIT markers of small dimensions or low Z, but still visible in CT and kV are needed. Promising results in terms of field perturbation were obtained for the carbon-coated markers and thin gold markers. Anchor markers and gold markers > 0.5 mm thick are not recommended to be used in ion beam therapy. Nevertheless, these markers could be considered for use lateral or distal to the treatment field. The known problems of fiducial migration, deformation, provoked streak artifacts, dose perturbations and the value of the WEPL will need to be evaluated in further studies before implementation in clinical routine.

## FUNDING

This work was funded by the ‘Klinische Forschergruppe Schwerionentherapie KFO 214’ and the Sonderforschungsbereich SFB/TRR 125, Project C01. Part of the analysis was supported by the European Community through the Seventh Framework Program, 2007–2012 PARTNER Project, (Grant Agreement No. 215840-2).
